# Modular Synthesis of Polyphenolic Benzofurans, and Application in the Total Synthesis of Malibatol A and Shoreaphenol

**DOI:** 10.3390/molecules15095909

**Published:** 2010-08-27

**Authors:** David Y.-K. Chen, Qiang Kang, T. Robert Wu

**Affiliations:** Chemical Synthesis Laboratory at Biopolis, Institute of Chemical and Engineering Sciences (ICES), Agency for Science, Technology and Research (A*STAR), 11 Biopolis Way, The Helios Block, No. 03-08, 138667, Singapore

**Keywords:** cascade reaction, polyphenol, resveratrol, Friedel-Crafts, natural product, total synthesis

## Abstract

A modular strategy for the synthesis of hexacyclic dimeric resveratrol polyphenolic benzofurans is reported. The developed synthetic technology was applied to the total synthesis of malibatol A, shoreaphenol, and other biologically relevant poly-phenols.

## 1. Introduction

**Figure 1:**
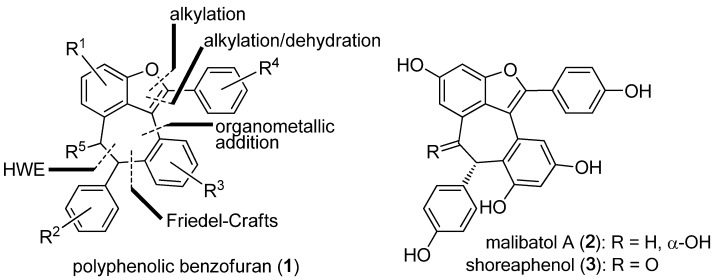
Generic molecular structure of polyphenolic benzofuran **1 **and structures of malibatol A (**2**) and shoreaphenol (**3**).

Polyphenolic secondary metabolites have attracted growing interest from the scientific community in recent years [1,2,3,4,5,6]. However, despite their fascinating molecular architectures and diverse biological properties, chemical syntheses of these natural products and/or designed analogues have been scarce [7,8]. With this in mind, and as a continuation of our chemical and biological investigations of polyphenolic natural products [9,10], we set out to develop a general strategy for the synthesis of dimeric, resveratrol-derived benzofurans represented by generic structure **1**, as shown in Figure 1. We further demonstrated the developed technology in the total synthesis of malibatol A (**2**) and shoreaphenol (**3**), two dimeric resveratrol polyphenolic benzofurans isolated from *Hopea malibato * and *Shorea robusta*, respectively [11,12,13].

## 2. Results and Discussion

**Scheme 1:**
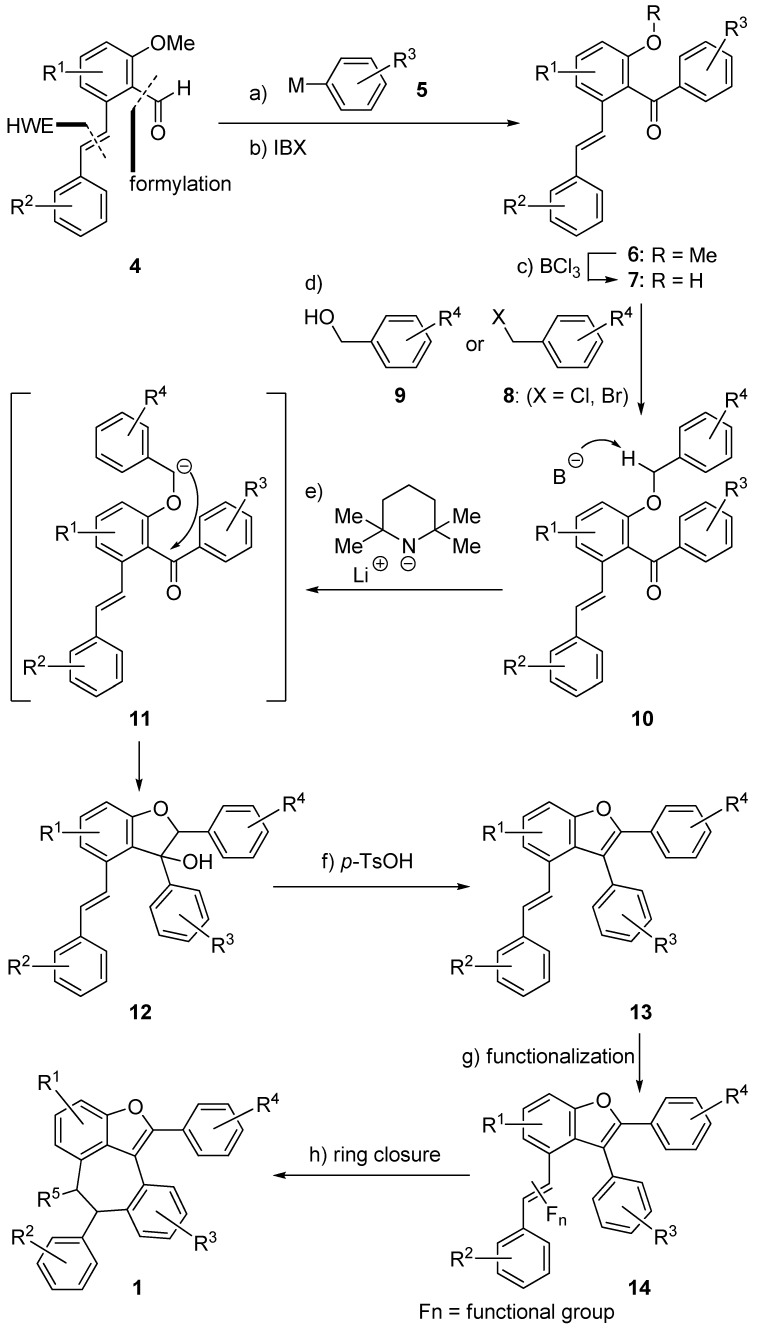
General, modular strategy for the construction of hexacyclic benzofuran **1**

Recognizing the hexacyclic structure represented by **1** containing four substituted phenyl rings, we envisaged a modular approach where each one of the phenyl rings can be installed independently and sequentially. Therefore, as outlined in Scheme 1, the proposed synthesis begin with stilbene aldehyde **4**, a building block with two aromatic domains brought together through a Horner-Wadswoth-Emmons (HWE) olefination reaction [14] and a subsequent Vilsmeier formylation [15]. Introduction of a third aromatic domain through the addition of an organometallic aryl species **5** to aldehyde **4**, followed by subsequent oxidation (IBX) should give ketone **6**. The intermediate benzylic alcohol obtained prior to IBX oxidation has previously been demonstrated by Snyder and co-workers as a versatile intermediate to access a number of resveratrol derived natural products [7,8]. Carbonyl-directed selective demethylation of **6 **should lead to phenol **7**, setting the stage for the attachment of the final aromatic moiety through an alkylation with benzyl halide **8** or a Mitsunobu reaction [16] with benzyl alcohol **9**. With benzyl ether **10** in hand, the formation of the benzofuran ring is anticipated through its initial benzylic deprotonation (LiTMP), followed by an intramolecular cyclization (**11** to **12**) and subsequent dehydration (**12** to **13**, *p*-TsOH•H_2_O), to deliver pentacyclic benzofuran **13** [17]. Finally, the olefinic functionality in stilbene **13 **should serve as a versatile handle for either direct seven-membered ring formation, or further transformation (**14**, e.g. epoxidation) leading to functionalized hexacycles **1 **upon ring closure.

With this general strategy in mind, its realization to generate a library of benzofuran polyphenols is illustrated in Table 1, Table 2 and Table 3. As shown in Table 1, aryl ketones **16** and benzyl ethers **17** were efficiently prepared in 85−90% yield (over the two steps from **15**) and 71−95% yield (over the two steps from **16**), respectively. Next, benzofuran formation from keto benzyl ethers **17** under the two-step procedure generally proceeded in good yields (71−85% yield, Table 2), apart from the failure of *p*-bromo substrate to participate in the cyclization (entry 3, Table 2) and the less satisfactory dehydration for the acid sensitive furanyl substrate (entry 5, Table 2).

**Table 1:** Preparation of ketone **16** and benzyl ether **17**.

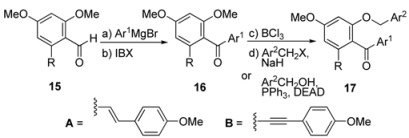					
Entry	R	Ar^1^	Ar^2^	16 Yield (%) *^b^*	17 Yield (%) *^b^*
1(a)	H	C_6_H_5_	C_6_H_5_	85%	89%
2(b)	A	3,5-(MeO)_2_C_6_H_3_	C_6_H_5_	88%	95%
3(c)	A	3,5-(MeO)_2_C_6_H_3_	4-(Br)C_6_H_4_	88%	92%
4(d)	A	3,5-(MeO)_2_C_6_H_3_	4-(MeO)C_6_H_4_	88%	90%
5(e)*^a^*	A	3,5-(MeO)_2_C_6_H_3_	2-furyl	88%	71%
6(f)	A	C_6_H_5_	4-(MeO)C_6_H_4_	85%	91%
7(g)	A	3,4,5-(MeO)_3_C_6_H_2_	4-(MeO)C_6_H_4_	90%	95%
8(h)	A	3,4-(MeO)_2_C_6_H_3_	4-(MeO)C_6_H_4_	87%	90%
9(i)	B	3,5-(MeO)_2_C_6_H_3_	4-(MeO)C_6_H_4_	86%	87%

*Reagents and conditions:* (a) Ar^1^MgBr (1.5 equiv), THF, 0 °C, 0.5 h; (b) IBX (2.0 equiv), DMSO, 23 °C, 2 h; (c) BCl_3_ (1.0 M in CH_2_Cl_2_, 1.5 equiv), CH_2_Cl_2_, 0 °C, 1 h; (d) NaH (2.0 equiv), Ar^2^CH_2_X (entry 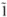
3, X = Br; entry 4, 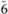
9, X = Cl; 1.4 equiv), DMF, 0 °C. *^a^*furanyl alcohol (3.0 equiv), PPh_3_ (3.0 equiv), DEAD (3.0 equiv), THF, 0 → 23 °C, 12 h. *^b^*Yields refer to chromatographically and spectroscopically homogeneous material. DMF = *N*,*N*'-dimethylformamide, IBX = *o*-iodoxybenzoic acid; DEAD = diethyl azodicarboxylate.

**Table 2:** Preparation of benzofuran **19**.

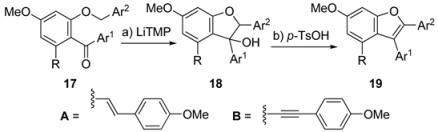				
Entry	R	Ar^1^	Ar^2^	19 Yield (%)*^a^*
1(a)	H	C_6_H_5_	C_6_H_5_	83
2(b)	A	3,5-(MeO)_2_C_6_H_3_	C_6_H_5_	71
3(c)	A	3,5-(MeO)_2_C_6_H_3_	4-(Br)C_6_H_4_	0
4(d)	A	3,5-(MeO)_2_C_6_H_3_	4-(MeO)C_6_H_4_	87
5(e)	A	3,5-(MeO)_2_C_6_H_3_	2-furyl	38
6(f)	A	C_6_H_5_	4-(MeO)C_6_H_4_	80
7(g)	A	3,4,5-(MeO)_3_C_6_H_2_	4-(MeO)C_6_H_4_	85
8(h)	A	3,4-(MeO)_2_C_6_H_3_	4-(MeO)C_6_H_4_	81
9(i)	B	3,5-(MeO)_2_C_6_H_3_	4-(MeO)C_6_H_4_	85

*Reagents and conditions:* (a) LiTMP (0.5 M in THF, 5 equiv), THF, 0 °C, 2 h; (b) *p*-TsOH•H_2_O (1.0 equiv), CH_2_Cl_2_, 23 °C, 1 h.*^ a^*Yields refer to chromatographically and spectroscopically homogeneous material. LiTMP = Lithium 2,2,6,6-tetramethylpiperidide; *p*-TsOH = toluenesulfonic acid.

Finally, closure of the seven-membered ring was carried out under acidic conditions (*p*-TsOH•H_2_O) to give cyclized compound **20** in high yields (90−95% yield, entries 1, 2, 5−7, Table 3). The incompatibility of the furanyl functionality under the acidic conditions was once again observed (entry 3, Table 3), and the electronically less favoured substrate **19d** failed to participate in the Friedel−Crafts type cyclization (entry 4, Table 3).

**Table 3:** Friedel−Crafts type cyclization of benzofurans **20**

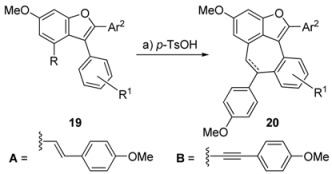				
Entry	R	R^1^	Ar^2^	20 Yield (%) *^a^*
1(a)	A	3,5-(MeO)_2_	C_6_H_5_	95
2(b)	A	3,5-(MeO)_2_	4-(MeO)C_6_H_4_	90
3(c)	A	3,5-(MeO)_2_	2-furyl	0
4(d)	A	H	4-(MeO)C_6_H_4_	0
5(e)	A	3,4,5-(MeO)_3_	4-(MeO)C_6_H_4_	92
6(f)	A	3,4-(MeO)_2_	4-(MeO)C_6_H_4_	90
7(g)	B	3,4-(MeO)_2_	4-(MeO)C_6_H_4_	95

*Reagents and conditions:* (a) *p*-TsOH•H_2_O (3.0 equiv), CH_2_Cl_2_, 40 °C, 8 h.*^ a^*Yields refer to chromatographically and spectroscopically homogeneous material.

**Scheme 2:**
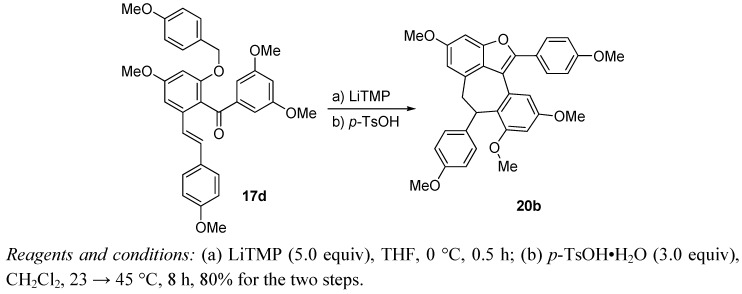
One-pot preparation of hexacyclic benzofuran **20b**.

In addition, we demonstrated a one-pot procedure to prepare hexacyclic benzofuran **20b** directly from keto benzyl ether stilbene **17d** (Scheme 2). This highly efficient, cascade process involving deprotonation-cyclization (LiTMP), dehydration and Friedel−Crafts ring-closure (*p*-TsOH) illustrated the utility of the developed methodology in the synthesis of highly functionalized, polycyclic polyphenols, a useful structural class for both chemical and biological investigations.

**Scheme 3:**
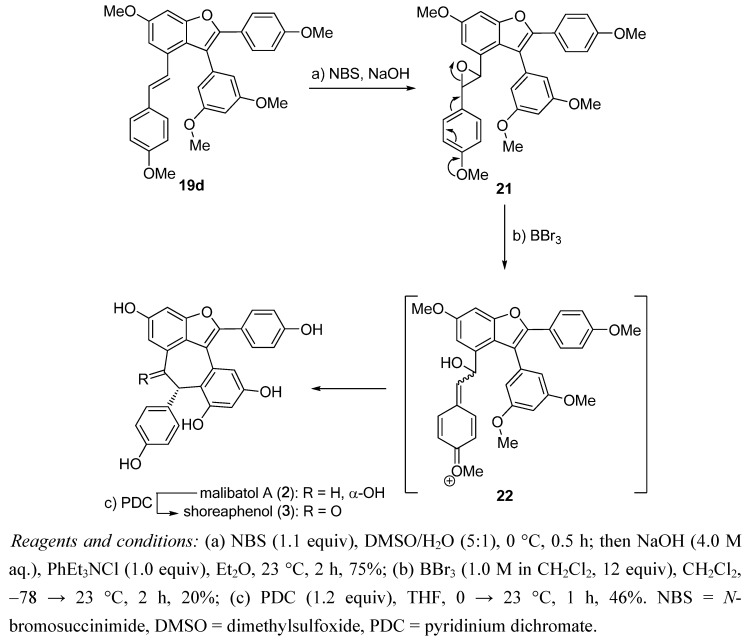
Total synthesis of malibatol A (**2**) and shoreaphenol (**3**).

Next, the developed methodology was applied to the total synthesis of malibatol A (**2**) [18] and shoreaphenol (**3**), as shown in Scheme 3 [19]. In this instance, with pentacyclic benzofuran **19d** in hand, construction of the oxygen-substituted, seven-membered ring in the malibatol A (**2**) and shoreaphenol (**3**) framework called for an intramolecular Friedel−Crafts type epoxide-opening process. Thus, epoxidation of stilbene **19d** under the bromohydrin protocol (NBS, NaOH), followed by treatment of the resulting epoxide (**21**) with BBr_3_ resulted the concomitant cyclization and global demethylation as a one-pot process, presumably through the intermediacy of **22**, giving racemic malibatol A (**2**) as a single diastereoisomer in 20% yield. Oxidation of malibatol A (**2**) in the presence of PDC then afforded shoreaphenol (**3**), despite the modest yield of 46%. Both malibatol A (**2**) and shoreaphenol (**3**) exhibited spectroscopic data (^1^H- and ^13^C-NMR) and mass spectrometry data matching those reported for the natural substances [11,12,13].

## 3. Experimental

### 3.1. General

All reactions were carried out under a nitrogen or argon atmosphere with dry solvents under anhydrous conditions, unless otherwise noted. Dry tetrahydrofuran (THF) and methylene chloride (CH_2_Cl_2_) were obtained by passing commercially available pre-dried, oxygen-free formulations through activated alumina columns. Methanol (MeOH), *N*,*N'*-dimethylformamide (DMF), dimethyl-sulfoxide (DMSO) and benzene were purchased in anhydrous form and used without further purification. Acetone, water, ethyl acetate (EtOAc), diethyl ether (Et_2_O), methylene chloride (CH_2_Cl_2_), and hexanes were purchased at the highest commercial quality and used without further purification, unless otherwise stated. Reagents were purchased at the highest commercial quality and used without further purification, unless otherwise stated. Yields refer to chromatographically and spectroscopically (^1^H-NMR) homogeneous materials, unless otherwise stated. Reactions were monitored by thin-layers chromatography (TLC) carried out on 0.25 mm E. Merck silica gel plates (60F-254) using UV light as visualizing agent and an ethanolic solution of ammonium molybdate and anisaldehyde and heat as developing agents. E. Merck silica gel (60, particle size 0.040−0.063 mm) was used for flash column chromatography. ^1^H and ^13^C-NMR spectra were recorded at 600 and 150 MHz, respectively, on a Bruker AV-600 instrument and calibrated using residual undeuterated solvent as an internal reference. The following abbreviations were used to explain the multiplicities: s = singlet, d = doublet, t = triplet, q = quartet, quint = quintet, m = multiplet, pent = pentet, hex = hexet, br = broad. IR spectra were recorded on a Perkin-Elmer Spectrum One FTIR spectrometer with diamond ATR accessory. Melting points (m.p.) are uncorrected and were recorded on a Buchi B-540 melting point apparatus. High-resolution mass spectra (HRMS) were recorded on an Agilent ESI TOF (time of flight) mass spectrometer at 3500 V emitter voltage.

### 3.2. General procedure A (Preparation of diaryl ketones ***16***, Table 1)

To a solution of aldehyde **15** (2.0 mmol) in THF (20 mL) at 0 °C was added the appropriate Grignard reagent (0.5 M in THF, 3.0 mmol). The resulting mixture was stirred for 0.5 h before it was quenched with NH_4_Cl (5 mL, sat. aq.). The layers were separated and the aqueous layer was extracted with EtOAc (3 × 10 mL). The combined organic layers were washed with brine (10 mL), dried (Na_2_SO_4_) and concentrated *in vacuo* to afford the crude benzyl alcohol, which was used directly without further purification. To the solution of crude benzyl alcohol (obtained as above) in DMSO (5 mL) at 23 °C was added IBX (1.15 g, 4.1 mmol) in one portion. The resulting mixture was stirred for 2 h before it was quenched with Na_2_S_2_O_3_ (5 mL, sat. aq.). The layers were separated and the aqueous layer was extracted with Et_2_O (3 × 30 mL). The combined organic layers were washed with brine (30 mL), dried (Na_2_SO_4_) and concentrated *in vacuo.* Flash column chromatography (silica gel) afforded diaryl ketone **16**. Using this general procedure the following compounds were prepared:

*(2,4-Dimethoxyphenyl)(phenyl)methanone* (**16a**). From 2,4-dimethoxybenzaldehyde and phenyl-magnesium bromide**.** Flash column chromatography (silica gel, hexanes-EtOAc 4:1) afforded ketone **16a** (412 mg, 85%) as a pale yellow foam. All physical properties of this compound were identical to those reported in literature [20].

*(E)-(2,4-Dimethoxy-6-(4-methoxystyryl)phenyl)(3,5-dimethoxyphenyl)methanone* (**16b**). From (*E*)-2,4-dimethoxy-6-(4-methoxystyryl)benzaldehyde and 3,5-dimethoxyphenylmagnesium bromide**.** Flash column chromatography (silica gel, hexanes-EtOAc 2:1) afforded ketone **16b** (764 mg, 88%) as a pale yellow foam. All physical properties of this compound were identical to those reported in literature [8].

*(E)-(2,4-Dimethoxy-6-(4-methoxystyryl)phenyl)(phenyl)methanone* (**16f**). From (*E*)-2,4-dimethoxy-6-(4-methoxystyryl)benzaldehyde and phenylmagnesium bromide. Flash column chromatography (silica gel, hexanes-EtOAc 2:1) afforded ketone **16f** (636 mg, 85%) as a pale yellow foam. **16f**: *R*_f_ = 0.45 (silica gel, hexanes-EtOAc 2:1); IR (film) ν_max_ 2938, 1661, 1595, 1510, 1253, 1161, 1078, 920, 830, 721 cm^−^^1^; ^1^H-NMR (CD_3_CN): δ = 7.80−7.78 (m, 2 H), 7.58−7.55 (m, 1 H), 7.46−7.43 (m, 2 H), 7.25 (d, *J* = 9.0 Hz, 2 H), 7.13 (d, *J* = 16.2 Hz, 1 H), 6.97 (d, *J* = 1.8 Hz, 1 H), 6.81 (d, *J* = 9.0 Hz, 2 H), 6.70 (d, *J* = 16.2 Hz, 1 H), 6.56 (d, *J* = 1.8 Hz, 1 H), 3.89 (s, 3 H), 3.71 (s, 3 H), 3.63 ppm (s, 3 H); ^13^C-NMR (CD_3_OD): δ = 197.2, 161.6, 159.7, 158.3, 138.3, 137.4, 133.5, 131.2, 129.4, 129.1, 128.7, 127.9, 122.4, 121.0, 114.1, 101.4, 97.7, 55.5, 55.3, 54.9 ppm; HRMS (ESI): calcd for C_24_H_22_O_4_Na^+^ [M + Na^+^] 397.1410, found 397.1406.

*(E)-(2,4-Dimethoxy-6-(4-methoxystyryl)phenyl)(3,4,5-trimethoxyphenyl)methanone* (**16g**). From (*E*)-2,4-dimethoxy-6-(4-methoxystyryl)benzaldehyde and 3,4,5-trimethoxyphenylmagnesium bromide. Flash column chromatography (silica gel, hexanes-EtOAc 2:1) afforded ketone **16g** (836 mg, 90%) as a pale yellow foam. **16g**: *R*_f_ = 0.25 (silica gel, hexanes-EtOAc 2:1); IR (film) ν_max_ 2938, 1661, 1578, 1511, 1413, 1327, 1156, 1126, 834 cm^−^^1^; ^1^H-NMR (CD_3_CN): δ = 7.28 (d, *J* = 8.4 Hz, 2 H), 7.13 (d, *J* = 16.2 Hz, 1 H), 7.08 (s, 2 H), 6.95 (d, *J* = 2.4 Hz, 1 H), 6.84 (d, *J* = 8.4 Hz, 2 H), 6.67 (d, *J* = 16.2 Hz, 1 H), 6.56 (d, *J* = 2.4 Hz, 1 H), 3.89 (s, 3 H), 3.77 (s, 3 H), 3.75 (s, 6 H), 3.74 (s, 3 H), 3.68 ppm (s, 3 H); ^13^C-NMR (CD_3_OD): δ = 195.8, 161.5, 159.7, 158.2, 153.3, 142.7, 137.5, 133.6, 131.1, 129.4, 127.9, 122.6, 120.8, 114.1, 106.6, 101.4, 97.7, 60.0, 55.7, 55.5, 55.3, 54.9 ppm; HRMS (ESI): calcd for C_27_H_28_O_7_Na^+^ [M + Na^+^] 487.1727, found 487.1712.

*(E)-(2,4-dimethoxy-6-(4-methoxystyryl)phenyl)(3,4-dimethoxyphenyl)methanone* (**16h**). From (*E*)-2,4-dimethoxy-6-(4-methoxystyryl)benzaldehyde and 3,4-dimethoxyphenylmagnesium bromide. Flash column chromatography (silica gel, hexanes-EtOAc 2:1) afforded ketone **16h** (755 mg, 87%) as a pale yellow foam. **16h**: *R*_f_ = 0.20 (silica gel, hexanes-EtOAc 2:1); IR (film) ν_max_ 2937, 1739, 1653, 1595, 1511, 1267, 1158, 835 cm^−^^1^; ^1^H-NMR (CD_3_CN): δ = 7.50 (d, *J* = 1.8 Hz, 1 H), 7.26 (d, *J* = 9.0 Hz, 2 H), 7.20 (dd, *J* = 8.4, 2.4 Hz, 1 H), 7.13 (d, *J* = 16.2 Hz, 1 H), 6.94 (d, *J* = 2.4 Hz, 1 H), 6.90 (d, *J* = 8.4 Hz, 1 H), 6.83 (d, *J* = 9.0 Hz, 2 H), 6.65 (d, *J* = 16.2 Hz, 1 H), 6.55 (d, *J* = 1.8 Hz, 1 H), 3.89 (s, 3 H), 3.84 (s, 3 H), 3.83 (s, 3 H), 3.74 (s, 3 H), 3.67 ppm (s, 3 H); ^13^C-NMR (CD_3_CN): δ = 195.5, 161.3, 159.7, 158.0, 153.8, 149.2, 137.1, 131.3, 130.9, 129.4, 127.8, 125.1, 122.5, 121.3, 114.1, 110.5, 110.1, 101.1, 97.7, 55.5, 55.5, 55.3, 55.2, 54.9 ppm; HRMS (ESI): calcd for C_26_H_26_O_6_Na^+^ [M + Na^+^] 457.1621, found 457.1610.

*(2,4-Dimethoxy-6-((4-methoxyphenyl)ethynyl)phenyl)(3,5-dimethoxyphenyl)methanone* (**16i**). From 2,4-dimethoxy-6-[(4-methoxyphenyl)ethynyl]benzaldehyde and 3,5-dimethoxyphenylmagnesium bromide. Flash column chromatography (silica gel, hexanes-EtOAc 2:1) afforded ketone **16i** (743 mg, 86%) as a pale yellow foam. **16i**: *R*_f_ = 0.28 (silica gel, hexanes-EtOAc 2:1); IR (film) ν_max_ 2938, 1671, 1590, 1569, 1510, 1247, 1153, 1065, 832 cm^−^^1^; ^1^H- NMR (CD_3_CN): δ = 7.10 (d, *J* = 9.0 Hz, 2 H), 6.92 (d, *J* = 2.4 Hz, 2 H), 6.84 (d, *J* = 9.0 Hz, 2 H), 6.73 (d, *J* = 2.4 Hz, 1 H), 6.72 (t, *J* = 2.4 Hz, 1 H), 6.66 (d, *J* = 2.4 Hz, 1 H), 3.86 (s, 3 H), 3.77 (s, 6 H), 3.75 (s, 3 H), 3.72 ppm (s, 3 H); ^13^C-NMR (CD_3_CN): δ = 195.7, 162.3, 162.0, 161.0, 158.8, 140.7, 133.6, 125.0, 123.2, 115.0, 114.8, 108.6, 107.7, 106.1, 100.3, 94.2, 86.4, 56.5, 56.3, 56.2, 55.9 ppm; HRMS (ESI): calcd for C_26_H_24_O_6_Na^+^ [M + Na^+^] 455.1465, found 455.1467.

### 3.3. General procedure B (Preparation of benzyl ethers ***17***, Table 1)

To a solution of diaryl ketone **16** (1.0 mmol) in CH_2_Cl_2_ (10 mL) at 0 °C was added BCl_3_ (1.0 M in CH_2_Cl_2_, 1.5 mL, 1.5 mmol) dropwise. The resulting mixture was stirred for 1 h before it was quenched with NH_4_Cl (10 mL, sat. aq.). The layers were separated and the aqueous layer was extracted with CH_2_Cl_2_ (3 × 10 mL). The combined organic layers were washed with brine (20 mL), dried (Na_2_SO_4_) and concentrated in *vacuo* to afford the crude phenol, which was used directly without further purification. To a solution of the crude phenol (obtained as above) in DMF (5 mL) at 0 °C was added NaH (80 mg, 60% wt/wt in mineral oil, 2.0 mmol). The resulting mixture was stirred for 0.5 h before benzyl bromide (or chloride) (1.4 mmol) was added. The reaction mixture was warmed to 23 °C and the progress was monitored by TLC analysis. Upon completion of the reaction (<4 h for most cases), the reaction mixture was quenched with NH_4_Cl (20 mL, sat. aq.). The layers were separated and the aqueous layer was extracted with Et_2_O (3 × 20 mL). The combined organic layers were washed with brine (30 mL), dried (Na_2_SO_4_) and concentrated in *vacuo*. Flash column chromatography (silica gel) afforded the desired benzyl ether **17**. Using the described general procedure the following substances were prepared:

*(2-(Benzyloxy)-4-methoxyphenyl)(phenyl)methanone* (**17a**). From ketone **16a** and benzyl bromide. Flash column chromatography (silica gel, hexanes-EtOAc 4:1) afforded benzyl ether **17a** (283 mg, 89%) as a yellow foam. **17a**: *R*_f_ = 0.35 (silica gel, hexanes-EtOAc 4:1); IR (film) ν_max_ 1651, 1601, 1579, 1501, 1446, 1272, 1166, 1120, 737, 697 cm^−^^1^; ^1^H-NMR (CD_3_CN): δ = 7.73−7.72 (m, 2 H), 7.57−7.55 (m, 1 H), 7.46−7.41 (m, 3 H), 7.21−7.16 (m, 3 H), 6.93−6.92 (m, 2 H), 6.69 (d, *J* = 2.4 Hz, 1 H), 6.64 (dd, *J* = 8.4, 2.4 Hz, 1 H), 4.97 (s, 2 H), 3.84 ppm (s, 3 H); ^13^C-NMR (CD_3_CN): δ = 195.6, 163.4, 158.3, 139.2, 136.5, 132.5, 131.6, 129.2, 128.3, 128.2, 127.6, 126.9, 121.7, 105.6, 99.7, 69.8, 55.4 ppm; HRMS (ESI): calcd for C_21_H_18_O_3_Na^+^ [M + Na^+^] 341.1148, found 341.1156.

*(E)-(2-(Benzyloxy)-4-methoxy-6-(4-methoxystyryl)phenyl)(3,5-dimethoxyphenyl)methanone* (**17b**). From ketone **16b** and benzyl bromide. Flash column chromatography (silica gel, hexanes-EtOAc 2:1) afforded benzyl ether **17b** (485 mg, 95%) as a yellow foam. **17b**: *R*_f_ = 0.45 (silica gel, hexanes-EtOAc 2:1); IR (film) ν_max_ 1667, 1594, 1511, 1301, 1204, 1156, 1065, 829 cm^−^^1^; ^1^H-NMR (CD_3_CN): δ = 7.31 (d, *J* = 9.0 Hz, 2 H), 7.24−7.23 (m, 3 H), 7.14 (d, *J* = 16.2 Hz, 1 H), 7.04−7.02 (m, 2 H), 6.96 (d, *J* = 2.4 Hz, 1 H), 6.88 (d, *J* = 2.4 Hz, 2 H), 6.86 (d, *J* = 9.0 Hz, 2 H), 6.73−6.70 (m, 2 H), 6.59 (d, *J* = 1.8 Hz, 1 H), 4.99 (s, 2 H), 3.88 (s, 3 H), 3.76 (s, 3 H), 3.75 ppm (s, 6 H); ^13^C-NMR (CD_3_CN): δ = 196.8, 161.5, 161.1, 159.8, 157.2, 140.8, 137.7, 136.6, 131.3, 129.4, 128.3, 127.9, 127.8, 127.2, 122.4, 121.3, 114.1, 106.7, 105.1, 101.7, 99.0, 70.0, 55.3, 55.3, 54.9 ppm; HRMS (ESI): calcd for C_32_H_30_O_6_Na^+^ [M + Na^+^] 533.1934, found 533.1951.

*(E)-(2-((4-Bromobenzyl)oxy)-4-methoxy-6-(4-methoxystyryl)phenyl)(3,5-dimethoxyphenyl)methanone* (**17c**). From ketone **16b** and 4-bromobenzyl bromide. Flash column chromatography (silica gel, hexanes-EtOAc 2:1) afforded benzyl ether **17c** (542 mg, 92%) as a yellow foam. **17c**: *R*_f_ = 0.41 (silica gel, hexanes-EtOAc 2:1); IR (film) ν_max_ 2837, 1666, 1593, 1510, 1300, 1156, 1066, 806 cm^−^^1^; ^1^H- NMR (CD_3_CN): δ = 7.37 (d, *J* = 8.4 Hz, 2 H), 7.31 (d, *J* = 9.0 Hz, 2 H), 7.15 (d, *J* = 16.2 Hz, 1 H), 6.97 (d, *J* = 2.4 Hz, 1 H), 6.93 (d, *J* = 8.4 Hz, 2 H), 6.86 (d, *J* = 9.0 Hz, 2 H), 6.84 (s, 2 H), 6.72 (d, *J* = 16.2 Hz, 1 H), 6.70 (t, *J* = 2.4 Hz, 1 H), 6.58 (d, *J* = 2.4 Hz, 1 H), 4.95 (s, 2 H), 3.88 (s, 3 H), 3.75 (s, 3 H), 3.74 ppm (s, 6 H); ^13^C-NMR (CD_3_CN): δ = 196.8, 161.5, 161.1, 159.8, 157.1, 140.9, 137.9, 135.9, 131.3, 131.3, 129.4, 129.1, 127.9, 122.3, 121.3, 121.1, 114.1, 106.6, 105.1, 101.9, 99.0, 69.3, 55.3, 55.3, 54.9 ppm; HRMS (ESI): calcd for C_32_H_29_BrO_6_Na^+^ [M + Na^+^] 611.1039, found 611.1033.

*(E)-(3,5-Dimethoxyphenyl)(4-methoxy-2-((4-methoxybenzyl)oxy)-6-(4-methoxystyryl)phenyl)-methan-one* (**17d**). From ketone **16b** and 4-methoxybenzyl chloride. Flash column chromatography (silica gel, hexanes-EtOAc 4:1) afforded benzyl ether **17d** (486 mg, 90%) as a pale yellow solid. **17d**: *R*_f_ = 0.40 (silica gel, hexanes-EtOAc 2:1); m.p. = 118−119 °C (hexanes-EtOAc); IR (film) ν_max_ 2970, 1738, 1594, 1512, 1352, 1302, 1249, 1204, 1156, 1065, 834 cm^−1^; ^1^H-NMR (CDCl_3_): δ = 7.31 (d, *J* = 9.0 Hz, 2 H), 7.03 (d, *J* = 15.0 Hz, 1 H), 6.98 (d, *J* = 2.4 Hz, 2 H), 6.92 (d, *J* = 9.0 Hz, 2 H), 6.86 (d, *J* = 2.4 Hz, 1 H), 6.85 (d, *J* = 14.4 Hz, 1 H), 6.82 (d, *J* = 9.0 Hz, 2 H), 6.74 (d, *J* = 9.0 Hz, 2 H), 6.66 (t, *J* = 2.4 Hz, 1 H), 6.45 (d, *J* = 2.4 Hz, 1 H), 4.88 (s, 2 H), 3.87 (s, 3 H), 3.77 (s, 6 H), 3.76 (s, 3 H), 3.75 ppm (s, 3 H); ^13^C-NMR (CDCl_3_): δ = 197.3, 161.1, 160.6, 159.3, 158.9, 157.3, 140.9, 137.8, 130.8, 129.4, 128.4, 128.2, 127.9, 122.8, 121.6, 113.8, 113.4, 106.9, 105.3, 101.2, 98.9, 69.8, 55.4, 55.3, 55.1, 55.0 ppm; HRMS (ESI): calcd for C_33_H_32_O_7_Na^+^ [M + Na^+^] 563.2040, found 563.2037.

*(E)-(3,5-Dimethoxyphenyl)(2-(furan-2-ylmethoxy)-4-methoxy-6-(4-methoxystyryl)phenyl)methanone* (**17e**). To a solution of phenol **16b** (420 mg, 1.0 mmol) in THF (10 mL) at 23 °C was added PPh_3_ (786 mg, 3.0 mmol). The resulting mixture was cooled to 0 °C before a solution of DEAD (522 mg, 3.0 mmol) and furfuryl alcohol (294 mg, 3.0 mmol) in THF (2 mL) were added. The resulting mixture was warmed to 23 °C and stirred for 12 h before it was quenched with NH_4_Cl (5 mL, sat. aq.). The layers were separated and the aqueous layer was extracted with EtOAc (3 × 10 mL). The combined organic layers were washed with brine (10 mL), dried (Na_2_SO_4_) and concentrated *in vacuo*. Flash column chromatography (silica gel, hexanes-EtOAc 4:1) afforded furanyl ether **17e** (355 mg, 71%) as a yellow oil. **17e**: *R*_f_ = 0.72 (silica gel, benzene-EtOAc 8:1); IR (film) ν_max_ 2937, 1667, 1592, 1510, 1456, 1300, 1155, 1063, 819 cm^−1^; ^1^H-NMR (CD_3_CN): δ = 7.38 (d, *J* = 1.2 Hz, 1 H), 7.28 (d, *J* = 9.0 Hz, 2 H), 7.13 (d, *J* = 16.2 Hz, 1 H), 6.97 (d, *J* = 2.4 Hz, 1 H), 6.85 (d, *J* = 9.0 Hz, 2 H), 6.82 (d, *J* = 2.4 Hz, 2 H), 6.69 (t, *J* = 2.4 Hz, 1 H), 6.68 (d, *J* = 2.4 Hz, 1 H), 6.65 (t, *J* = 16.2 Hz, 1 H), 6.33−6.32 (m, 1 H), 6.28 (d, *J* = 3.6 Hz, 1 H), 4.95 (s, 2 H), 3.90 (s, 3 H), 3.75 (s, 3 H), 3.74 ppm (s, 6 H); ^13^C-NMR (CD_3_CN): δ = 197.4, 162.2, 161.9, 160.6, 157.6, 150.7, 144.2, 141.3, 138.5, 132.2, 130.2, 128.8, 123.1, 122.3, 115.0, 111.3, 111.1, 107.6, 106.0, 103.0, 100.1, 63.5, 56.2, 56.1, 55.8 ppm; HRMS (ESI): calcd for C_30_H_28_O_7_Na^+^ [M + Na^+^] 523.1727, found 523.1725.

*(E)-(4-Methoxy-2-((4-methoxybenzyl)oxy)-6-(4-methoxystyryl)phenyl)(phenyl)methanone* (**17f**). From ketone **16f** and 4-methoxybenzyl chloride. Flash column chromatography (silica gel, hexanes-EtOAc 2:1) afforded benzyl ether **17f** (437 mg, 91%) as a yellow foam. **17f**: *R*_f_ = 0.50 (silica gel, hexanes-EtOAc 2:1); IR (film) ν_max_ 1661, 1595, 1511, 1249, 1163, 1033, 827, 721 cm^−^^1^; ^1^H-NMR (CD_3_CN): δ = 7.77−7.75 (m, 2 H), 7.62−7.59 (m, 1 H), 7.47 (t, *J* = 7.8 Hz, 2 H), 7.28 (d, *J* = 9.0 Hz, 2 H), 7.14 (d, *J* = 16.2 Hz, 1 H), 6.97 (d, *J* = 1.8 Hz, 1 H), 6.89 (d, *J* = 8.4 Hz, 2 H), 6.83 (d, *J* = 9.0 Hz, 2 H), 6.74 (d, *J* = 8.4 Hz, 2 H), 6.72 (d, *J* = 16.2 Hz, 1 H), 6.61 (d, *J* = 1.8 Hz, 1 H), 4.89 (s, 2 H), 3.89 (s, 3 H), 3.74 (s, 3 H), 3.71 ppm (s, 3 H); ^13^C-NMR (CD_3_CN): δ = 198.5, 162.8, 161.0, 160.6, 158.7, 139.9, 139.0, 134.7, 132.5, 130.7, 130.4, 130.3, 130.0, 129.7, 129.2, 123.7, 122.8, 115.4, 114.9, 103.0, 100.4, 71.2, 56.6, 56.2, 56.1 ppm; HRMS (ESI): calcd for C_31_H_28_O_5_Na^+^ [M + Na^+^] 503.1829, found 503.1817.

*(E)-(4-Methoxy-2-((4-methoxybenzyl)oxy)-6-(4-methoxystyryl)phenyl)(3,4,5-trimethoxyphenyl)methan-one* (**17g**). From ketone **16g** and 4-methoxybenzyl chloride. Flash column chromatography (silica gel, hexanes-EtOAc 2:1) afforded benzyl ether **17g** (542 mg, 95%) as a yellow foam. **17g**: *R*_f_ = 0.30 (silica gel, hexanes-EtOAc 2:1); IR (film) ν_max_ 1655, 1595, 1512, 1413, 1327, 1249, 1157, 1126, 832 cm^−^^1^; ^1^H-NMR (*d*_6_-acetone): δ 7.35 (d, *J* = 9.0 Hz, 2 H), 7.22 (d, *J* = 15.6 Hz, 1 H), 7.08 (s, 2 H), 7.05 (d, *J* = 2.4 Hz, 1 H), 6.97 (d, *J* = 8.4 Hz, 2 H), 6.88 (d, *J* = 8.4 Hz, 2 H), 6.85 (d, *J* = 15.6 Hz, 1 H), 6.77 (d, *J* = 8.4 Hz, 2 H), 6.68 (d, *J* = 2.4 Hz, 1 H), 4.99 (s, 2 H), 3.91 (s, 3 H), 3.81 (s, 3 H), 3.77 (s, 9 H), 3.74 ppm (s, 3 H); ^13^C-NMR (*d*_6_-acetone): δ = 195.3, 161.4, 159.8, 159.3, 157.5, 153.4, 142.8, 137.7, 134.4, 130.8, 129.6, 128.7, 128.6, 127.9, 122.7, 121.6, 114.0, 113.4, 106.6, 101.4, 99.0, 69.6, 59.8, 55.6, 54.9, 54.6, 54.5 ppm; HRMS (ESI): calcd for C_34_H_34_O_8_Na^+^ [M + Na^+^] 593.2145, found 593.2137.

*(E)-(3,4-Dimethoxyphenyl)(4-methoxy-2-((4-methoxybenzyl)oxy)-6-(4-methoxystyryl)phenyl)methan-one* (**17h**). From ketone **16h** and 4-methoxybenzyl chloride. Flash column chromatography (silica gel, hexanes-EtOAc 2:1) afforded benzyl ether **17h** (486 mg, 90%) as a yellow foam. **17h**: *R*_f_ = 0.25 (silica gel, hexanes-EtOAc 2:1); IR (film) ν_max_ 1652, 1594, 1510, 1265, 1249, 1159, 1121, 1024, 833 cm^−^^1^; ^1^H-NMR (CD_3_CN): δ 7.45 (d, *J* = 1.8 Hz, 1 H), 7.27 (d, *J* = 9.0 Hz, 2 H), 7.20 (dd, *J* = 8.4, 1.8 Hz, 1 H), 7.13 (d, *J* = 16.2 Hz, 1 H), 6.98 (d, *J* = 8.4 Hz, 2 H), 6.95 (d, *J* = 2.4 Hz, 1 H), 6.89 (d, *J* = 8.4 Hz, 1 H), 6.83 (d, *J* = 9.0 Hz, 2 H), 6.76 (d, *J* = 9.0 Hz, 2 H), 6.70 (d, *J* = 16.2 Hz, 1 H), 6.60 (d, *J* = 2.4 Hz, 1 H), 4.91 (s, 2 H), 3.87 (s, 3 H), 3.83 (s, 3 H), 3.80 (s, 3 H), 3.73 (s, 3 H), 3.72 ppm (s, 3 H); ^13^C-NMR (CD_3_CN): δ = 195.7, 161.2, 159.7, 159.3, 157.1, 153.8, 149.2, 137.3, 131.6, 130.9, 129.4, 129.0, 128.5, 127.8, 124.9, 122.5, 121.9, 114.1, 113.6, 110.5, 110.2, 101.4, 99.2, 69.9, 55.6, 55.3, 55.3, 54.9, 54.8 ppm; HRMS (ESI): calcd for C_33_H_32_O_7_Na^+^ [M + Na^+^] 563.2040, found 563.2057.

*(3,5-Dimethoxyphenyl)(4-methoxy-2-((4-methoxybenzyl)oxy)-6-((4-methoxyphenyl)ethynyl)phenyl)methanone* (**17i**). From ketone **16i** and 4-methoxybenzyl chloride. Flash column chromatography (silica gel, hexanes-EtOAc 2:1) afforded benzyl ether **17i** (468 mg, 87%) as a yellow foam. **17i**: *R*_f_ = 0.27 (silica gel, hexanes-EtOAc 2:1); IR (film) ν_max_ 2937, 1671, 1591, 1511, 1300, 1248, 1155, 1063, 832 cm^−^^1^; ^1^H -NMR (CD_3_CN): δ = 7.12 (d, *J* = 8.4 Hz, 2 H), 7.07 (d, *J* = 9.0 Hz, 2 H), 6.88 (d, *J* = 2.4 Hz, 2 H), 6.84 (d, *J* = 9.0 Hz, 2 H), 6.81 (d, *J* = 8.4 Hz, 2 H), 6.74 (d, *J* = 2.4 Hz, 1 H), 6.72 (t, *J* = 2.4 Hz, 1 H), 6.71 (d, *J* = 2.4 Hz, 1 H), 4.97 (s, 2 H), 3.85 (s, 3 H), 3.77 (s, 6 H), 3.76 (s, 3 H), 3.74 ppm (s, 3 H); ^13^C-NMR (CD_3_CN): δ = 195.8, 162.2, 162.0, 161.0, 160.3, 157.8, 140.9, 133.6, 130.0, 129.1, 125.5, 123.4, 115.0, 114.8, 114.5, 108.9, 107.6, 106.0, 101.8, 94.1, 86.4, 70.9, 56.3, 56.2, 55.9, 55.7 ppm; HRMS (ESI): calcd for C_33_H_30_O_7_Na^+^ [M + Na^+^] 561.1883, found 561.1883.

### 3.4. General procedure C (Preparation of benzofurans ***19***, Table 2)

To a solution of benzyl ether **17** (0.2 mmol) in THF (2 mL) at 0 °C was added LiTMP (0.5 M in THF, 2 mL, 1.0 mmol). The resulting mixture was stirred at 0 °C and the progress was monitored by TLC analysis. Upon completion of the reaction (~ 2 h for most cases), the reaction mixture was quenched with NH_4_Cl (5 mL, sat. aq.). The layers were separated and the aqueous layer was extracted with EtOAc (3 × 5 mL). The combined organic layers were washed with brine (10 mL), dried (Na_2_SO_4_) and concentrated *in vacuo* to afford crude tertiary alcohol **18**, which was used directly without further purification. To a solution of the crude tertiary alcohol **18** (obtained as above) in CH_2_Cl_2_ (3 mL) at 23 °C was added *p*-TsOH•H_2_O (38 mg, 0.2 mmol). The resulting mixture was stirred for 1 h before it was quenched with NaHCO_3_ (3 mL, sat. aq.). The layers were separated and the aqueous layer was extracted with CH_2_Cl_2_ (3 × 5 mL). The combined organic layers were washed with brine (5 mL), dried (Na_2_SO_4_) and concentrated *in vacuo.* Flash column chromatography (silica gel) afforded the desired benzofuran **19**. Using this general procedure the following compounds were prepared:

*6-Methoxy-2,3-diphenylbenzofuran* (**19a**). From benzyl ether **17a**. Flash column chromatography (silica gel, hexanes-EtOAc 4:1) afforded benzofuran **19a** (50 mg, 83%) as a pale yellow oil. All physical properties of this compound were identical to those reported in literature [21].

*(E)-3-(3,5-Dimethoxyphenyl)-6-methoxy-4-(4-methoxystyryl)-2-phenylbenzofuran* (**19b**). From benzyl ether **17b**. Flash column chromatography (silica gel, hexanes-EtOAc 4:1) afforded benzofuran **19b** (70 mg, 71%) as a yellow oil. **19b**: *R*_f_ = 0.42 (silica gel, hexanes-EtOAc 2:1); IR (film) ν_max_ 1601, 1510, 1420, 1249, 1204, 1143, 1064, 1033, 808, 693 cm^−^^1^; ^1^H-NMR (CD_3_CN): δ = 7.58−7.57 (m, 2 H), 7.32−7.25 (m, 3 H), 7.15 (d, *J* = 2.4 Hz, 1 H), 7.06 (d, *J* = 2.4 Hz, 1 H), 7.01 (d, *J* = 16.2 Hz, 1 H), 6.99 (d, *J* = 8.4 Hz, 2 H), 6.83 (d, *J* = 9.0 Hz, 2 H), 6.79 (d, *J* = 16.2 Hz, 1 H), 6.72 (t, *J* = 2.4 Hz, 1 H), 6.65 (d, *J* = 2.4 Hz, 2 H), 3.88 (s, 3 H), 3.77 (s, 3H), 3.74 ppm (s, 6 H); ^13^C-NMR (CD_3_CN): δ = 163.1, 160.8, 159.9, 156.3, 150.6, 138.0, 133.6, 131.9, 131.2, 130.1, 129.8, 129.3, 128.8, 127.1, 123.4, 122.8, 119.3, 115.3, 109.9, 108.0, 101.0, 96.1, 56.8, 56.6, 56.3 ppm; HRMS (ESI): calcd for C_32_H_28_O_5_Na^+^ [M + Na^+^] 515.1829, found 515.1837.

*(E)-2-(4-Bromophenyl)-3-(3,5-dimethoxyphenyl)-6-methoxy-4-(4-methoxystyryl)benzofuran* (**19c**). From benzyl ether **17c**. The desired benzofuran **19c** was not obtained in this reaction.

*(E)-3-(3,5-Dimethoxyphenyl)-6-methoxy-2-(4-methoxyphenyl)-4-(4-methoxystyryl)benzofuran* (**19d**). From benzyl ether **17d**. Flash column chromatography (silica gel, hexanes:-EtOAc 2:1) afforded benzofuran **19d** (91 mg, 87%) as a yellow solid. **19d**: *R*_f_ = 0.52 (silica gel, hexanes-EtOAc 2:1); m.p. = 61−62 °C (hexanes-EtOAc); IR (film) ν_max_ 2936, 1603, 1509, 1250, 1153, 1143, 1033, 833, 808 cm^−^^1^; ^1^H-NMR (CD_3_CN): δ = 7.43 (d, *J* = 8.4 Hz, 2 H), 7.07 (d, *J* = 2.4 Hz, 1 H), 6.95−6.89 (m, 4 H), 6.78−6.75 (m, 5 H), 6.68 (t, *J* = 2.4 Hz, 1 H), 6.59 (d, *J* = 2.4 Hz, 2 H), 3.82 (s, 3 H), 3.73 (s, 3 H), 3.70 (s, 6 H), 3.69 ppm (s, 3 H); ^13^C-NMR (CD_3_CN): δ = 161.6, 159.4, 159.3, 158.1, 154.8, 149.4, 137.0, 131.8, 130.0, 128.4, 127.4, 127.3, 123.1, 122.4, 121.7, 116.2, 113.9, 113.8, 108.7, 106.4, 99.5, 94.7, 55.3, 55.2, 54.9, 54.8 ppm; HRMS (ESI): calcd for C_33_H_30_O_6_Na^+^ [M + Na^+^] 545.1934, found 545.1951.

*(E)-3-(3,5-Dimethoxyphenyl)-2-(furan-2-yl)-6-methoxy-4-(4-methoxystyryl)benzofuran* (**19e**). From furanyl ether **17e**. Flash column chromatography (silica gel, hexanes-EtOAc 2:1) afforded benzofuran **19e** (36 mg, 38%) as a yellow oil. **19e**: *R*_f_ = 0.40 (silica gel, hexanes-EtOAc 2:1); IR (film) ν_max_ 2936, 1600, 1510, 1421, 1250, 1152, 1064, 819 cm^−1^; ^1^H-NMR (CD_3_CN): δ = 7.50 (d, *J* = 1.2 Hz, 1 H), 7.16 (d, *J* = 2.4 Hz, 1 H), 7.06 (d, *J* = 2.4 Hz, 1 H), 7.02 (d, *J* = 15.6 Hz, 1 H), 7.01 (d, *J* = 8.4 Hz, 2 H), 6.84−6.82 (m, 3 H), 6.70 (t, *J* = 2.4 Hz, 1 H), 6.62 (d, *J* = 2.4 Hz, 2 H), 6.46 (q, *J* = 1.8 Hz, 1 H), 6.38 (d, *J* = 3.0 Hz, 1 H), 3.88 (s, 3 H), 3.77 (s, 3 H), 3.76 ppm (s, 6 H); ^13^C-NMR (CD_3_CN): δ = 162.2, 160.4, 159.5, 156.1, 146.2, 143.9, 143.4, 136.3, 133.1, 130.7, 129.8, 128.3, 123.0, 121.5, 114.9, 112.5, 109.4, 107.7, 100.6, 95.8, 56.4, 56.1, 55.8 ppm; HRMS (ESI): calcd for C_30_H_26_O_6_Na^+^ [M + Na^+^] 505.1621, found 505.1603.

*(E)-6-Methoxy-2-(4-methoxyphenyl)-4-(4-methoxystyryl)-3-phenylbenzofuran* (**19f**). According to General Procedure C using benzyl ether **17f**. Flash column chromatography (silica gel, hexanes-EtOAc 4:1) afforded benzofuran **19f** (74 mg, 80%) as a yellow oil. **19f**: *R*_f_ = 0.60 (silica gel, hexanes-EtOAc 2:1); IR (film) ν_max_ 1605, 1511, 1251, 1175, 1144, 1033, 833, 702 cm^−1^; ^1^H-NMR (*d*_6_-acetone): δ = 7.62−7.59 (m, 3 H), 7.52−7.50 (m, 2 H), 7.45 (d, *J* = 9.0 Hz, 2 H), 7.18 (d, *J* = 2.4 Hz, 1 H), 7.09 (d, *J* = 2.4 Hz, 1 H), 7.03 (d, *J* = 16.2 Hz, 1 H), 6.96 (d, *J* = 8.4 Hz, 2 H), 6.86 (d, *J* = 9.0 Hz, 2 H), 6.80 (d, *J* = 8.4 Hz, 2 H), 6.73 (d, *J* = 16.2 Hz, 1 H), 3.91 (s, 3 H), 3.77 ppm (s, 6 H); ^13^C-NMR (*d*_6_-acetone): δ = 159.5, 159.5, 158.4, 155.0, 149.7, 135.1, 131.9, 130.7, 129.9, 129.3, 128.6, 128.0, 127.6, 127.3, 123.2, 121.9, 121.8, 116.4, 113.8, 113.8, 106.5, 94.7, 55.2, 54.7, 54.6 ppm; HRMS (ESI): calcd for C_31_H_26_O_4_Na^+^ [M + Na^+^] 485.1723, found 485.1713. 

*(E)-6-Methoxy-2-(4-methoxyphenyl)-4-(4-methoxystyryl)-3-(3,4,5-trimethoxyphenyl)benzofuran* (**19g**). According to General Procedure C using benzyl ether **17g**. Flash column chromatography (silica gel, hexanes:EtOAc 2:1) afforded benzofuran **19g** (94 mg, 85%) as a yellow oil. **19g**: *R*_f_ = 0.34 (silica gel, hexanes:EtOAc 2:1); IR (film) ν_max_ 2936, 1604, 1511, 1409, 1250, 1126, 1033, 838 cm^−^^1^; ^1^H-NMR (CD_3_CN): δ = 7.50 (d, *J* = 9.0 Hz, 2 H), 7.14 (d, *J* = 1.8 Hz, 1 H), 7.05−6.99 (m, 4 H), 6.86 (d, *J* = 9.0 Hz, 2 H), 6.81 (d, *J* = 16.2 Hz, 1 H), 6.78 (d, *J* = 9.0 Hz, 2 H), 6.75 (s, 2 H), 3.88 (s, 3 H), 3.86 (s, 3 H), 3.76 (s, 3 H), 3.75 (s, 3 H), 3.68 ppm (s, 6 H); ^13^C-NMR (CD_3_CN): δ = 159.5, 159.4, 158.2, 154.8, 154.0, 149.7, 137.9, 132.0, 130.1, 130.0, 128.8, 127.4, 127.3, 123.2, 122.3, 121.7, 116.4, 114.0, 113.9, 107.9, 106.6, 94.8, 60.3, 55.9, 55.4, 54.9, 54.9 ppm; HRMS (ESI): calcd for C_34_H_32_O_7_Na^+^ [M + Na^+^] 575.2040, found 575.2048.

*(E)-3-(3,4-Dimethoxyphenyl)-6-methoxy-2-(4-methoxyphenyl)-4-(4-methoxystyryl)benzofuran* (**19h**). From benzyl ether **17h**. Flash column chromatography (silica gel, hexanes-EtOAc 2:1) afforded benzofuran **19h** (85 mg, 81%) as a yellow oil. **19h**: *R*_f_ = 0.35 (silica gel, hexanes-EtOAc 2:1); IR (film) ν_max_ 2923, 1604, 1509, 1247, 1138, 1026, 833, 734 cm^−^^1^; ^1^H-NMR (CD_3_CN): δ = 7.49 (d, *J* = 9.0 Hz, 2 H), 7.12 (d, *J* = 1.8 Hz, 1 H), 7.08 (d, *J* = 8.4 Hz, 1 H), 7.03 (d, *J* = 2.4 Hz, 1 H), 7.02 (d, *J* = 2.4 Hz, 1 H), 6.99−6.96 (m, 4 H), 6.85 (d, *J* = 9.0 Hz, 2 H), 6.79 (d, *J* = 9.0 Hz, 2 H), 6.73 (d, *J* = 16.2 Hz, 1 H), 3.92 (s, 3 H), 3.88 (s, 3 H), 3.77 (s, 3 H), 3.76 (s, 3 H), 3.67 ppm (s, 3 H); ^13^C-NMR (CD_3_CN): δ = 159.4, 159.4, 158.2, 154.8, 149.9, 149.8, 149.3, 132.0, 130.0, 128.5, 127.5, 127.3, 126.9, 123.3, 122.9, 122.3, 122.0, 116.3, 114.2, 113.9, 113.9, 112.2, 106.4, 94.8, 55.6, 55.5, 55.4, 54.9 ppm; HRMS (ESI): calcd for C_33_H_30_O_6_Na^+^ [M + Na^+^] 545.1934, found 545.1946.

*3-(3,5-Dimethoxyphenyl)-6-methoxy-2-(4-methoxyphenyl)-4-((4-methoxyphenyl)ethynyl)benzofuran* (**19i**). From benzyl ether **17i**. Flash column chromatography (silica gel, hexanes-EtOAc 4:1) afforded benzofuran **19i** (88 mg, 85%) as a yellow oil. **19i**: *R*_f_ = 0.70 (silica gel, hexanes-EtOAc 2:1); IR (film) ν_max_ 2935, 1604, 1510, 1485, 1248, 1152, 1035, 831 cm^−^^1^; ^1^H-NMR (CD_3_CN): δ = 7.47 (d, *J* = 9.0 Hz, 2 H), 7.16 (d, *J* = 2.4 Hz, 1 H), 7.02 (d, *J* = 8.4 Hz, 2 H), 6.99 (d, *J* = 2.4 Hz, 1 H), 6.87 (d, *J* = 9.0 Hz, 2 H), 6.85 (d, *J* = 8.4 Hz, 2 H), 6.64 (d, *J* = 2.4 Hz, 2 H), 6.56 (t, *J* = 2.4 Hz, 1 H), 3.87 (s, 3 H), 3.79 (s, 3 H), 3.77 (s, 3 H), 3.70 ppm (s, 6 H); ^13^C-NMR (CD_3_CN): δ = 161.8, 160.7, 160.6, 158.5, 155.4, 151.5, 135.8, 133.7, 128.6, 123.9, 123.7, 117.3, 116.4, 116.0, 115.5, 114.8, 114.7, 109.9, 100.4, 97.6, 94.6, 86.1, 56.5, 55.9, 55.8 ppm; HRMS (ESI): calcd for C_33_H_28_O_6_Na^+^ [M + Na^+^] 543.1778, found 543.1773. 

### 3.5. General procedure D (Preparation of hexacyclic benzofurans ***20***, Table 3)

To a solution of benzofuran **19** (0.04 mmol) in CH_2_Cl_2_ (6 mL) at 23 °C was added *p*-TsOH•H_2_O (22.8 mg, 0.12 mmol). The resulting mixture was heated to 40 °C and stirred for 8 hours before it was quenched with NaHCO_3_ (3 mL, sat. aq.). The layers were separated and the aqueous layer was extracted with CH_2_Cl_2_ (3 × 5 mL). The combined organic layers were washed with brine (5 mL), dried (Na_2_SO_4_) and concentrated in *vacuo.* Flash column chromatography (silica gel) afforded the desired hexacyclic benzofuran **20**. The following compounds were prepared *via* this general procedure:

*1,3,8-Trimethoxy-11-(4-methoxyphenyl)-5-phenyl-10,11-dihydrobenzo[6,7]cyclohepta[1,2,3-cd]benzo-furan* (**20a**). From benzofuran **19b**. Flash column chromatography (silica gel, hexanes-EtOAc 4:1) afforded hexacyclic benzofuran **20a** (18.7 mg, 95%) as a yellow oil. **20a**: *R*_f_ = 0.57 (silica gel, hexanes-EtOAc 2:1); IR (film) ν_max_ 2933, 1598, 1509, 1461, 1248, 1144, 1065, 853, 696 cm^−^^1^; ^1^H- NMR (CD_3_CN): δ = 7.66−7.64 (m, 2 H), 7.47−7.41 (m, 3 H), 7.01 (d, *J* = 8.4 Hz, 2 H), 6.78 (d, *J* = 1.8 Hz, 1 H), 6.71 (d, *J* = 1.8 Hz, 1 H), 6.59 (d, *J* = 8.4 Hz, 2 H), 6.58 (d, *J* = 2.4 Hz, 1 H), 6.53 (d, *J* = 2.4 Hz, 1 H), 5.44 (d, *J* = 5.4 Hz, 1 H), 3.81 (s, 3 H), 3.78 (s, 3 H), 3.72 (dd, *J* = 16.2, 5.4 Hz, 1 H), 3.58 (s, 3 H), 3.44 (s, 3 H), 3.37 ppm (d, *J* = 16.2 Hz, 1 H); ^13^C-NMR (CD_3_CN): δ = 158.6, 158.3, 158.1, 157.2, 154.2, 150.6, 135.4, 134.1, 133.9, 131.7, 128.9, 128.7, 128.7, 128.3, 123.9, 119.8, 112.9, 112.6, 107.2, 97.8, 92.7, 55.8, 55.2, 54.5, 54.4, 36.7, 36.6 ppm; HRMS (ESI): calcd for C_32_H_28_O_5_Na^+^ [M + Na^+^] 515.1829, found 515.1835.

*1,3,8-Trimethoxy-5,11-bis(4-methoxyphenyl)-10,11-dihydrobenzo[6,7]cyclohepta[1,2,3-cd]benzofuran* (**20b**). From benzofuran **19d**. Flash column chromatography (silica gel, hexanes-EtOAc 4:1) afforded hexacyclic benzofuran **20b** (18.8 mg, 90%) as a yellow oil. **20b**: *R*_f_ = 0.49 (silica gel, hexanes-EtOAc 2:1); IR (film) ν_max_ 2933, 1599, 1508, 1460, 1248, 1143, 1067, 1032, 834 cm^−^^1^; ^1^H-NMR (CD_3_CN): δ = 7.57 (d, *J* = 9.0 Hz, 2 H), 7.00−6.98 (m, 4 H), 6.75 (d, *J* = 1.8 Hz, 1 H), 6.69 (d, *J* = 1.8 Hz, 1 H), 6.61 (d, *J* = 2.4 Hz, 1 H), 6.58 (d, *J* = 9.0 Hz, 2 H), 6.51 (d, *J* = 2.4 Hz, 1 H), 5.43 (d, *J* = 6.0 Hz, 1 H), 3.83 (s, 3 H), 3.80 (s, 3 H), 3.76 (s, 3 H), 3.69 (dd, *J* = 16.2, 6.0 Hz, 1 H), 3.57 (s, 3 H), 3.47 (s, 3 H), 3.35 ppm (d, *J* = 16.2 Hz, 1 H); ^13^C-NMR (CD_3_CN): δ = 160.2, 158.6, 158.3, 157.8, 157.2, 154.0, 150.7, 135.2, 134.2, 134.1, 130.2, 128.3, 123.9, 123.8, 119.9, 116.1, 114.1, 112.9, 112.3, 106.9, 97.6, 92.6, 55.8, 55.2, 55.1, 54.5, 54.4, 36.7, 36.6 ppm; HRMS (ESI): calcd for C_33_H_30_O_6_Na^+^ [M + Na^+^] 545.1934, found 545.1948.

*5-(Furan-2-yl)-1,3,8-trimethoxy-11-(4-methoxyphenyl)-10,11-dihydrobenzo[6,7]cyclohepta[1,2,3-cd]-benzofuran* (**20c**). From benzofuran **19e**. The desired product **20c** was not obtained in this reaction.

*8-Methoxy-5,11-bis(4-methoxyphenyl)-10,11-dihydrobenzo[6,7]cyclohepta[1,2,3-cd]benzofuran* (**20d**). From benzofuran **19f**. The desired product **20d** was not obtained in this reaction.

*1,2,3,8-Tetramethoxy-5,11-bis(4-methoxyphenyl)-10,11-dihydrobenzo[6,7]cyclohepta[1,2,3-cd]benzo-furan* (**20e**). From benzofuran **19g**. Flash column chromatography (silica gel, hexanes-EtOAc 4:1) afforded hexacyclic benzofuran **20e** (20.3 mg, 92%) as a yellow oil. **20e**: *R*_f_ = 0.48 (silica gel, hexanes-EtOAc 2:1); IR (film) ν_max_ 2933, 1611, 1508, 1316, 1248, 1143, 1037, 835 cm^−^^1^; ^1^H-NMR (CD_3_CN): δ = 7.60 (d, *J* = 8.4 Hz, 2 H), 7.02−7.00 (m, 4 H), 6.89 (s, 1 H), 6.75 (s, 1 H), 6.69 (s, 1 H), 6.58 (d, *J* = 8.4 Hz, 2 H), 5.31 (d, *J* = 5.4 Hz, 1 H), 3.84 (s, 3 H), 3.83 (s, 3 H), 3.76 (s, 3 H), 3.74 (dd, *J* = 16.2, 5.4 Hz, 1 H), 3.73 (s, 3 H), 3.40 (d, *J* = 16.2 Hz, 1 H), 3.39 ppm (s, 3 H); ^13^C-NMR (CD_3_CN): δ = 160.2, 157.9, 157.2, 154.0, 152.2, 151.2, 150.1, 141.4, 134.9, 134.1, 130.1, 129.4, 128.3, 128.0, 123.9, 119.9, 115.8, 114.1, 112.9, 112.4, 110.2, 92.7, 61.2, 60.2, 55.2, 55.1, 54.8, 54.5, 38.0, 36.8 ppm; HRMS (ESI): calcd for C_33_H_30_O_6_Na^+^ [M + Na^+^] 545.1934, found 545.1948.

*2,3,8-Trimethoxy-5,11-bis(4-methoxyphenyl)-10,11-dihydrobenzo[6,7]cyclohepta[1,2,3-cd]benzofuran* (**20f**). From benzofuran **19h**. Flash column chromatography (silica gel, hexanes-EtOAc 2:1) afforded hexacyclic benzofuran **20f** (18.8 mg, 90%) as a yellow oil. **20f**: *R*_f_ = 0.35 (silica gel, hexanes-EtOAc 2:1); IR (film) ν_max_ 2934, 1610, 1511, 1499, 1251, 1177, 1143, 1035, 834, 793 cm^−^^1^; ^1^H-NMR (CD_3_CN): δ = 8.07 (d, *J* = 9.0 Hz, 2 H), 7.23 (s, 1 H), 7.08 (d, *J* = 9.0 Hz, 2 H), 6.86 (d, *J* = 8.4 Hz, 2 H), 6.70 (d, *J* = 8.4 Hz, 2 H), 6.64 (d, *J* = 2.4 Hz, 1 H), 6.57 (s, 1 H), 6.21 (d, *J* = 2.4 Hz, 1 H), 4.63 (dd, *J* = 6.6, 2.4 Hz, 1 H), 3.90 (s, 3 H), 3.74 (s, 3 H), 3.69 (s, 3 H), 3.66 (s, 3 H), 3.65 (s, 3 H), 3.62 (dd, *J* = 14.4, 2.4 Hz, 1 H), 3.15 ppm (dd, *J* = 14.4, 6.6 Hz, 1 H); ^13^C-NMR (CD_3_CN): δ = 193.6, 165.3, 165.0, 162.1, 158.8, 153.5, 150.5, 148.4, 139.9, 139.6, 136.3, 132.9, 131.8, 130.4, 127.8, 122.6, 114.9, 114.6, 114.1, 113.4, 112.7, 107.6, 56.3, 56.3, 56.2, 56.1, 55.6, 49.7, 41.4 ppm; HRMS (ESI): calcd for C_33_H_30_O_6_Na^+^ [M + Na^+^] 545.1934, found 545.1943.

*1,3,8-Trimethoxy-5,11-bis(4-methoxyphenyl)benzo[6,7]cyclohepta[1,2,3-cd]benzofuran* (**20g**). From benzofuran **19i**. Flash column chromatography (silica gel, hexanes-EtOAc 4:1) afforded hexacyclic benzofuran **20g** (19.8 mg, 95%) as a yellow oil. **20g**: *R*_f_ = 0.70 (silica gel, hexanes-EtOAc 2:1); IR (film) ν_max_ 2919, 1738, 1606, 1505, 1462, 1365, 1247, 1199, 833 cm^−^^1^; ^1^H-NMR (CD_3_CN): δ = 7.82 (d, *J* = 9.0 Hz, 2 H), 7.21 (d, *J* = 9.0 Hz, 2 H), 7.08 (d, *J* = 9.0 Hz, 2 H), 6.86 (d, *J* = 9.0 Hz, 2 H), 6.78 (d, *J* = 1.8 Hz, 1 H), 6.64 (d, *J* = 1.8 Hz, 1 H), 6.62 (d, *J* = 2.4 Hz, 1 H), 6.59 (s, 1 H), 6.29 (d, *J* = 2.4 Hz, 1 H), 3.86 (s, 3 H), 3.80 (s, 3 H), 3.78 (s, 3 H), 3.43 (s, 3 H), 3.17 ppm (s, 3 H); ^13^C-NMR (CD_3_CN): δ = 161.4, 161.4, 161.3, 159.8, 158.8, 155.0, 151.9, 141.9, 141.7, 138.2, 133.8, 132.4, 131.0, 127.4, 127.1, 125.0, 120.6, 116.4, 115.1, 114.1, 111.9, 106.2, 100.3, 94.6, 56.2, 56.0, 56.0, 55.7, 55.3 ppm; HRMS (ESI): calcd for C_33_H_30_O_6_Na^+^ [M + Na^+^] 543.1784, found 543.1745.

### 3.6. One-pot preparation of hexacyclic benzofuran ***20b***

To a solution of benzyl ketone **17d** (100 mg, 0.185 mmol) in THF (3 mL) at 0 °C was added LiTMP (0.5 M in THF, 1.9 mL, 0.93 mmol). The resulting mixture was stirred at 0 °C for 30 min before it was quenched with NH_4_Cl (5 mL, sat. aq.). The layers were separated and the aqueous layer was extracted with EtOAc (3 × 5 mL). The combined organic layers were washed with brine (5 mL), dried (Na_2_SO_4_) and concentrated *in vacuo* to afford crude alcohol, which was used directly without further purification. To a solution of the crude alcohol (obtained as above) in CH_2_Cl_2_ (3 mL) at 23 °C was added *p*-TsOH•H_2_O (105 mg, 0.56 mmol). The resulting mixture was heated to 45 °C and stirred for 8 h before it was quenched with NaHCO_3_ (3 mL, sat. aq.). The layers were separated and the aqueous layer was extracted with CH_2_Cl_2_ (3 × 5 mL). The combined organic layers were washed with brine (5 mL), dried (Na_2_SO_4_) and concentrated *in vacuo.* Flash column chromatography (silica gel, hexanes-EtOAc 2:1) afforded the desired hexacyclic benzofuran **20b **(77 mg, 80%) as a yellow oil.

*(E)-(3,5-Dimethoxyphenyl)(2-hydroxy-4-methoxy-6-(4-methoxystyryl)phenyl)methanone* (**16b'**). To a solution of ketone **16b** (13.0 g, 30 mmol) in CH_2_Cl_2_ (100 mL) at 0 °C was added BCl_3_ (1 M in CH_2_Cl_2_, 45 mL, 45 mmol) dropwise. The resulting mixture was stirred for 1 h before it was quenched with NaHCO_3 _(50 mL, sat. aq.). The layers were separated and the aqueous layer was extracted with CH_2_Cl_2_ (3 × 50 mL). The combined organic layers were washed with brine (100 mL), dried (Na_2_SO_4_) and concentrated *in vacuo*. Flash column chromatography (silica gel, hexanes-EtOAc-CH_2_Cl_2_ 4:1:1) afforded phenol **16b'** (12 g, 95%) as a yellow solid. **16b'**: *R*_f_ = 0.45 (silica gel, hexanes-EtOAc 2:1); m.p. = 139−140 °C (hexanes-EtOAc); IR (film) ν_max_ 2939, 1600, 1511, 1457, 1254, 1204, 1157, 1064, 840, 808 cm^−^^1^; ^1^H-NMR (CDCl_3_): δ = 11.5 (br s, 1 H), 6.89 (d, *J* = 8.4 Hz, 2 H), 6.74 (d, *J* = 8.4 Hz, 2 H), 6.72 (d, *J* = 2.4 Hz, 2 H), 6.67 (d, *J* = 2.4 Hz, 1 H), 6.65 (d, *J* = 16.2 Hz, 1 H), 6.48 (t, *J* = 2.4 Hz, 1 H), 6.47 (d, *J* = 2.4 Hz, 1 H), 6.46 (d, *J* = 15.6 Hz, 1 H), 3.88 (s, 3 H), 3.78 (s, 3 H), 3.68 ppm (s, 6 H); ^13^C-NMR (CDCl_3_): δ = 200.1, 164.7, 164.6, 160.6, 159.4, 142.7, 142.7, 130.0, 129.5, 127.7, 126.9, 113.8, 113.3, 106.9, 106.3, 104.4, 99.9, 55.6, 55.2 ppm; HRMS (ESI): calcd for C_25_H_24_O_6_Na^+^ [M + Na^+^] 443.1465, found 443.1454.

*3-(3,5-Dimethoxyphenyl)-6-methoxy-2-(4-methoxyphenyl)-4-(3-(4-methoxyphenyl)oxiran-2-yl)benzo-furan* (**21**). To a solution of benzofuran **19d** (4.20 g, 8.04 mmol) in DMSO (50 mL) and water (10 mL) at 0 °C was added NBS (1.57 g, 8.84 mmol) in one portion. The resulting mixture was stirred for 0.5 h before it was quenching with Na_2_S_2_O_3 _(50 mL, sat. aq.). The layers were separated and the aqueous layer was extracted with Et_2_O (3 × 100 mL). The combined organic layers were washed with brine (100 mL), dried (Na_2_SO_4_) and concentrated *in vacuo* to afford the crude bromohydrin, which was use directly without further purification. To a solution of crude bromohydrin (obtained as above) in Et_2_O (100 mL) at 23 °C were added NaOH (4 M, aq., 30 mL) and PhEt_3_NCl (1.83 g, 8.04 mmol). The resulting mixture was stirred for 2 h before the layers were separated, and the aqueous layer was extracted with Et_2_O (3 × 100 mL). The combined organic layers were washed with brine (100 mL), dried (Na_2_SO_4_) and concentrated *in vacuo*. Flash column chromatography (silica gel, hexanes-EtOAc 2:1) afforded epoxide **21** (3.25 g, 75%, over the two steps) as a yellow solid. **21**: *R*_f_ = 0.55 (silica gel, hexanes-EtOAc 2:1); m.p. = 147−148 °C (hexanes/CH_2_Cl_2_); IR (film) ν_max_ 2939, 1738, 1611, 1587, 1512, 1204, 1154, 1033, 832 cm^−^^1^; ^1^H-NMR (CDCl_3_): δ = 7.47 (d, *J* = 9.0 Hz, 2 H), 7.02 (d, *J* = 1.8 Hz, 1 H), 6.89 (d, *J* = 8.4 Hz, 2 H), 6.86 (d, *J* = 1.8 Hz, 1 H), 6.80 (d, *J* = 8.4 Hz, 2 H), 6.79 (d, *J* = 9.0 Hz, 2 H), 6.54 (br, 1 H), 6.31 (br, 1 H), 6.18 (t, *J* = 1.8 Hz, 1 H), 3.89 (s, 3 H), 3.83 (s, 3 H), 3.79 (d, *J* = 2.4 Hz, 1 H), 3.77 (s, 3 H), 3.74 (br, 3 H), 3.53 (d, *J* = 2.4 Hz, 1 H), 3.30 ppm (br, 3 H); ^13^C-NMR (CDCl_3_): δ = 161.0, 159.7, 159.2, 158.2, 154.2, 149.7, 136.0, 131.5, 128.5, 127.2, 126.9, 123.2, 122.5, 115.4, 113.8, 113.6, 107.7 (br), 105.9, 99.7, 95.3, 63.1, 59.1, 55.8, 55.3, 55.2, 54.7 (br) ppm; HRMS (ESI): calcd for C_33_H_30_O_7_Na^+^ [M + Na^+^] 561.1883, found 561.1898.

*Malibatol A* (**2**): To a solution of epoxide **21** (100 mg, 0.19 mmol) in CH_2_Cl_2_ (30 mL) at −78 °C was added BBr_3_ (1.0 M in CH_2_Cl_2_, 2.28 mL, 2.28 mmol). The resulting mixture was warmed to 23 °C and stirred for 2 h before it was quenched with NaHCO_3 _(10 mL, sat. aq.). The layers were separated and the aqueous layer was extracted with EtOAc (3 × 20 mL). The combined organic layers were washed with brine (20 mL), dried (Na_2_SO_4_) and concentrated *in vacuo*. Flash column chromatography (silica gel, CH_2_Cl_2_-MeOH 9:1) afforded malibatol (**2**, 17.8 mg, 20%) as a tan oil. Compound **2**: *R*_f_ = 0.23 (silica gel, CH_2_Cl_2_-MeOH 9:1); IR (film) ν_max_ 3323, 2918, 1612, 1510, 1433, 1366, 1231, 1139, 833 cm^−1^; ^1^H-NMR (CD_3_OD): δ = 7.45 (d, *J* = 8.6 Hz, 2 H), 7.02 (d, *J* = 8.6 Hz, 2 H), 7.01 (d, *J* = 2.4 Hz, 1 H), 6.80 (d, *J* = 8.6 Hz, 2 H), 6.57 (dd, *J* = 2.4, 1.2 Hz, 1 H), 6.51 (d, *J* = 2.4 Hz, 1 H), 6.33 (d, *J* = 9.0 Hz, 2 H), 6.30 (d, *J* = 2.4 Hz, 1 H), 5.46 (brs, 1 H), 5.28 ppm (m, 1 H); ^13^C-NMR (CD_3_OD): δ = 159.1, 157.4, 156.7, 156.2, 155.3, 155.1, 151.2, 139.6, 135.8, 133.4, 130.9, 130.6, 124.6, 121.2, 119.0, 117.3, 116.4, 114.7, 109.9, 109.7, 102.1, 95.9, 74.8, 48.9 ppm; HRMS (ESI): calcd for C_28_H_20_O_7_Na^+^ [M + Na^+^] 491.1101, found 491.1092.

*Shoreaphenol* (**3**): To a solution of malibatol A (**2**) (5 mg, 10.7 µmol) in THF (1 mL) at 23 °C was added PDC (4.8 mg, 12.8 µmol). The resulting mixture was stirred for 1 h before it was quenched with Na_2_S_2_O_3_ (1 mL, sat. aq.). The layers were separated and the aqueous layer was extracted with EtOAc (3 × 2 mL). The combined organic layers were washed with brine (3 mL), dried (Na_2_SO_4_) and concentrated *in vacuo*. Flash column chromatography (silica gel, CH_2_Cl_2_-MeOH 5:1) afforded shoreaphenol (**3**, 2.3 mg, 46%) as a yellow oil. Compound **3**: *R*_f_ = 0.26 (silica gel, CH_2_Cl_2_-MeOH 9:1); IR (film) ν_max_ 3339, 1738, 1612, 1366, 1216, 829 cm^−1^; ^1^H-NMR (*d*_6_-acetone): δ = 7.70 (d, *J* = 8.4 Hz, 2 H), 7.33 (d, *J* = 2.4 Hz, 1 H), 7.04 (d, *J* = 1.8 Hz, 1 H), 6.98 (d, *J* = 8.4 Hz, 2 H), 6.85 (d, *J* = 7.8 Hz, 2 H), 6.70 (d, *J* = 2.4 Hz, 1 H), 6.57 (d, *J* = 2.4 Hz, 1 H), 6.55 (d, *J* = 8.4 Hz, 2 H), 6.12 (brs, 1 H), 5.28 ppm (m, 1 H); ^13^C-NMR (*d*_6_-acetone): δ = 196.3, 159.5, 158.3, 157.7, 156.4, 156.1, 154.9, 153.3, 135.2, 131.1, 131.0, 130.6, 128.5, 123.1, 122.4, 116.6, 116.4, 115.6, 114.0, 112.0, 109.0, 103.0, 102.4, 56.1 ppm; HRMS (ESI): calcd for C_28_H_18_O_7_Na^+^ [M + Na^+^] 489.0950, found 489.0955.

## 4. Conclusions

In conclusion, a modular and efficient entry to the dimeric resveratrol derived polyphenolic benzofurans has been developed, and applied to the total synthesis of malibatol A (**2**) and shoreaphenol (**3**). In view of the largely untapped potential of the polyphenolic secondary metabolites, the synthetic methodology described herein should find wide application in the chemical and biological investigations of this fascinating class of compounds.
